# Ferroptosis in Pulmonary Disease and Lung Cancer: Molecular Mechanisms, Crosstalk Regulation, and Therapeutic Strategies

**DOI:** 10.1002/mco2.70116

**Published:** 2025-02-23

**Authors:** Dandan Guo, Songhua Cai, Lvdan Deng, Wangting Xu, Sentao Fu, Yaling Lin, Tong Jiang, Qing Li, Zhijun Shen, Jian Zhang, Peng Luo, Bufu Tang, Ling Wang

**Affiliations:** ^1^ The Department of Oncology First Affiliated Hospital of Dalian Medical University Dalian Liaoning China; ^2^ Department of Thoracic Surgery National Cancer Center National Clinical Research Center for Cancer Cancer Hospital & Shenzhen Hospital Chinese Academy of Medical Sciences and Peking Union Medical College Shenzhen Guangdong China; ^3^ Department of Respiratory First Affiliated Hospital School of Medicine Zhejiang University Hangzhou Zhejiang China; ^4^ The Department of Oncology Zhujiang Hospital Southern Medical University Guangzhou Guangdong China; ^5^ Department of Radiation Oncology Zhongshan Hospital Fudan University Shanghai Shanghai China

**Keywords:** ferroptosis, immune microenvironment, lung cancer, molecular mechanisms, pulmonary disease

## Abstract

Ferroptosis is a distinct form of iron‐dependent programmed cell death characterized primarily by intracellular iron accumulation and lipid peroxidation. Multiple cellular processes, including amino acid metabolism, iron metabolism, lipid metabolism, various signaling pathways, and autophagy, have been demonstrated to influence the induction and progression of ferroptosis. Recent investigations have elucidated that ferroptosis plays a crucial role in the pathogenesis of various pulmonary disorders, including lung injury, chronic obstructive pulmonary disease, pulmonary fibrosis, and asthma. Ferroptosis is increasingly recognized as a promising novel strategy for cancer treatment. Various immune cells within the tumor microenvironment, including CD8+ T cells, macrophages, regulatory T cells, natural killer cells, and dendritic cells, have been shown to induce ferroptosis in tumor cells and modulate the process through the regulation of iron and lipid metabolism pathways. Conversely, ferroptosis can reciprocally alter the metabolic environment, leading to the activation or inhibition of immune cell functions, thereby modulating immune responses. This paper reviews the molecular mechanism of ferroptosis and describes the tumor immune microenvironment, discusses the connection between ferroptosis and the tumor microenvironment in lung cancer and pulmonary diseases, and discusses the development prospect of their interaction in the treatment of lung cancer and pulmonary diseases.

## Graphical abstract

Ferroptosis, a cell death mode marked by iron accumulation and lipid peroxidation, plays a vital role in lung diseases and cancer. Its interaction with immune cells in the tumor microenvironment offers new therapeutic opportunities for lung cancer and pulmonary diseases

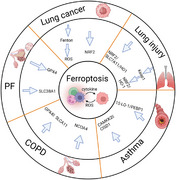

## 1 Introduction

**FIGURE 1:**
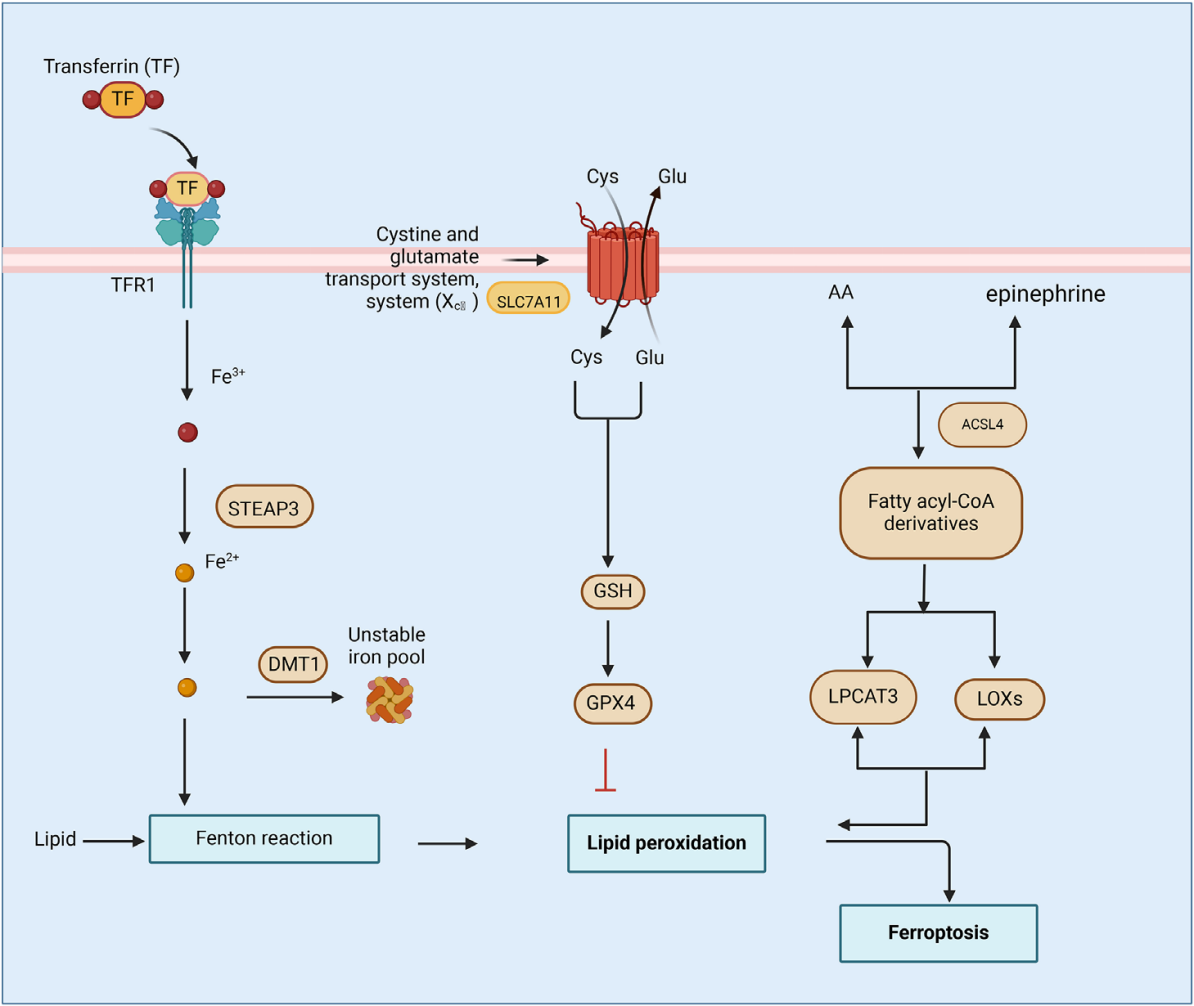
Diagram of regulatory mechanisms of ferroptosis. The classic ferroptosis signaling pathways (GSH metabolism, lipid metabolism, iron metabolism) differ in their metabolic pathways. This diagram illustrates how iron metabolism, lipid metabolism, and GSH metabolism lead to lipid peroxidation, causing or inhibiting ferroptosis. After iron enters the cell through transferrin, it participates in the Fenton reaction, generating free radicals that lead to lipid peroxidation. Glutathione (GSH) and GPX4 inhibit lipid peroxidation to prevent ferroptosis. Fatty acid metabolism (such as arachidonic acid) promotes lipid peroxidation through ACSL4 and LOXs, ultimately triggering ferroptosis.

When lipid peroxides reach excessive levels, they activate ferroptosis, a recently characterized form of cellular death. The hallmark of ferroptosis is characterized by the depletion of intracellular glutathione (GSH) and the inactivation of glutathione peroxidase 4 (GPX4), resulting in severe oxidative stress damage to cell membrane phospholipids. During this process, iron catalyzes the generation of free radicals through the Fenton reaction, thereby further accelerating lipid peroxidation. Ferroptosis has emerged as an approach with potential for tumor therapy, being regulated by GSH–GPX metabolism, lipid metabolism, and iron metabolism, with particular emphasis on the metabolism of unsaturated fatty acids. Ferroptosis has been associated with various diseases, particularly cancer, where tumor cells often evade cell death by suppressing ferroptosis. Conversely, targeting ferroptosis pathways serves as an innovative strategy in cancer treatment [1, 2, 3, 4]. Compared with apoptosis and other death modes, ferroptosis exhibits unique characteristics. The morphological features of ferroptotic cells include an intact cell membrane, a normal nucleus size, greater mitochondrial density, reduction or loss of cristae, and membrane shrinkage [5]. Key features of apoptosis include chromatin compaction and cellular shrinking, formation of membrane vesicles, nuclear fragmentation, generation of apoptotic bodies, and subsequent engulfment by phagocytes. Necrosis, in contrast, is characterized by cell swelling, membrane rupture, leakage of cellular contents, and the subsequent induction of inflammatory reactions. Pyroptosis exhibits morphological features such as cell swelling, pore development in the plasma membrane, subsequent membrane rupture, and release of cellular contents, ultimately leading to potent inflammatory reactions. During autophagic cell death, cells show increased autophagosomes and extensive organelle degradation, while the cell membrane typically remains intact [6, 7]. Iron metabolism and lipid peroxidation drive ferroptosis, making it distinct from apoptosis, necrosis, and autophagy. The distinctive feature of ferroptosis lies in its mechanism, which does not involve the typical organelle degradation processes observed in other cell death pathways. Various cell death pathways play distinct roles in physiologic and pathologic circumstances [8, 9, 10]. In the case of ferroptosis, its associated metabolic and signaling pathways are intricately linked to its initiation and progression [11, 12] (Figure 1). Research exploring the treatment opportunities using ferroptosis in disease treatment has emerged as a highly promising avenue in biomedical sciences.

The tumor microenvironment (TME) is a highly complex and dynamically evolving ecosystem. This intricate milieu encompasses the immediate vicinity of the tumor and comprises various components, including blood vessels, immune cells, fibroblasts, signaling molecules, and the extracellular matrix. TME plays a pivotal role in multiple aspects of tumor biology, including initiation, proliferation, angiogenesis, invasion, metastasis, and the development of treatment resistance. The tumor immune microenvironment (TIME), a critical component of the TME, specifically encompasses the immune cells and their complex interactions with tumor cells. The TIME comprises various immune cell populations, including but not limited to macrophages, T lymphocytes, dendritic cells (DCs), and natural killer (NK) cells, which collectively is essential for modulating immune escape, immune suppression, and the efficacy of tumor immunotherapy [13, 14, 15]. Immune cell survival and function in the TIME can be markedly altered by ferroptosis, thereby influencing the complex interactions and dynamics of the immune microenvironment. Consequently, the manipulation of ferroptosis has developed as a promising innovative method for treating various diseases, particularly in the context of cancer immunotherapy.

The mutual influence between the TIME and ferroptosis has emerged as a major emphasis in recent cancer research. Various components of the TIME, including immune cells, metabolic factors, and oxidative stress, may modulate the initiation of ferroptosis. Conversely, ferroptosis can reciprocally influence the TIME, potentially affecting tumor progression and treatment efficacy. Research has confirmed that ferroptosis significantly influences iron metabolism and lipid peroxidation, with significant implications for neurodegenerative diseases, cancer therapeutics, and acute organ injuries [16, 17, 18, 19, 20]. In an effort to expand the therapeutic arsenal against lung cancer and pulmonary diseases, we have conducted a comprehensive review of ferroptosis and TIME applications in these conditions. Additionally, we explored their potential in nanotechnology‐based interventions, aiming to provide novel avenues for clinical disease management.

## 2 Mechanism of Ferroptosis

Since its initial characterization by Dixon et al. in 2012 [21], research on ferroptosis has demonstrated exponential growth, emerging as a crucial field in cell death studies. A specific type of regulated cell death, ferroptosis occurs through iron‐dependent lipid reactive oxygen species (ROS) accumulation and GSH reduction, with the final outcome of lipid peroxide formation. Targeting this specific death pathway offers promising directions in cancer treatment [21]. Ferroptosis exists within a complex network of cellular processes and is tightly regulated by diverse metabolic pathways, including but not limited to amino acid, iron, and lipid metabolism [22]. Mechanistically, ferroptosis involves the sophisticated interaction of GSH metabolism, particularly through the GPX4‐dependent antioxidant system, lipid metabolism via acyl‐CoA synthase long chain member 4 (ACSL4)‐mediated lipid modifications, and iron metabolism through iron‐regulatory proteins. These regulatory networks are further modulated by disease‐specific signaling pathways, suggesting potential therapeutic implications across various pathological conditions.

### 2.1 GSH Metabolism

GSH, a tripeptide antioxidant composed of glutamic acid, cysteine, and glycine, plays a crucial role in maintaining immune system homeostasis [23, 24]. Functioning as an essential intracellular redox buffer, GSH executes multifaceted protective functions, including ROS detoxification, redox equilibrium maintenance, and cellular defense fortification against oxidative challenge. This versatile tripeptide transcends its classical antioxidant role, serving as a critical mediator in diverse biological processes, from cell cycle progression and apoptotic signaling to immune system regulation. The cystine/glutamate antiporter system (System Xc−) facilitates amino acid transport across cellular membranes. Under normal physiological conditions, System Xc− imports cystine into cells and exports glutamate out of cells at a 1:1 ratio [25, 26]. This stringently regulated exchange machinery orchestrates the precise equilibrium of cystine transport, establishing a robust pipeline for intracellular cysteine generation, an essential precursor process that safeguards GSH biosynthetic capacity under both homeostatic and stress‐challenged cellular conditions. The core component of this system, SLC7A11, is a transmembrane protein that regulates GSH synthesis and subsequently suppresses ferroptosis and oxidative stress. The biosynthesis of GSH proceeds through enzymatic catalysis, incorporating glycine, glutamic acid, and cysteine in a multistep process [27]. The inhibition of System Xc− decreases cysteine synthesis and subsequently reduces GSH synthesis, resulting in lipid peroxidation [28]. GPX4, a selenium‐containing protein, is a very important regulator of ferroptosis. GPX4 maintains lipid oxidation balance and defends cells against oxidative stress‐induced ferroptosis [29]. GSH, with GPX4 as a catalyst, works to decrease lipid peroxide generation and minimize oxidative stress‐induced damage. When GPX4 is inactivated, lipid peroxidation occurs, resulting in lipid peroxide accumulation [30, 31, 32]. That is, System Xc− inhibition or reduced GPX4 activity further promotes ferroptosis.

### 2.2 Lipid Metabolism

Lipid peroxidation is an oxidation chain reaction in which polyunsaturated fatty acids (PUFAs) become oxidized to form lipid peroxides [33, 34]. This process serves as a canonical hallmark of oxidative stress and orchestrates crucial functions across diverse physiological and pathological contexts, most notably in ferroptosis, a genetically encoded cell death modality identified by iron‐dependent lipid peroxidation cascades. PUFAs, by virtue of their molecular architecture featuring multiple unsaturated bonds, exhibit pronounced vulnerability to oxidative modifications, thereby functioning as preferential substrates in peroxidation reactions. The accumulation of PUFAs was found to be an important factor influencing the occurrence of ferroptosis. This selective accumulation serves dual regulatory functions: it establishes a critical pool of oxidation‐susceptible substrates while simultaneously modulating cellular vulnerability to oxidative insult, thereby functioning as a master determinant in the orchestration of ferroptosis pathways. The process of PUFAs accumulation is roughly as follows: arachidonic alcohol and epinephrine synthesize fatty acyl‐coenzyme A derivatives under the action of ACSL4, and they are oxidized to lipid peroxides by lysophosphatidylcholine acyl‐transferase 3 (LPCAT3) and lipoxygenase (LOXs) [35, 36, 37].ACSL4 executes a fundamental catalytic function in PUFA activation through stereospecific conversion to acyl‐CoA derivatives, which subsequently undergo LPCAT3‐mediated incorporation into membrane phospholipids. These PUFA‐enriched phospholipid domains exhibit exceptional susceptibility to peroxidation, whereby LOX‐mediated oxidative modification generates lipid hydroperoxides that compromise membrane architectural integrity and initiate ferroptotic signaling cascades. This precisely orchestrated molecular sequence underscores the essential regulatory functions of ACSL4, LPCAT3, and LOXs in governing both lipid peroxidation dynamics and ferroptotic cell fate decisions. ACSL4 and LPCAT3 are enzymes that drive lipid peroxide production, regulating ferroptosis activation [38, 39]. In this intricately coordinated cascade, ACSL4 functions as a critical determinant of oxidation‐susceptible PUFA bioavailability, while LPCAT3 orchestrates their precise incorporation into membrane phospholipids, collectively expanding the substrate repertoire for LOX‐mediated oxidative modification. This sophisticated enzymatic interplay exemplifies the exquisitely regulated nature of lipid peroxidation machinery and its profound implications in ferroptosis pathways. Lipid peroxidation is regulated by ACSL4, LPCAT3, and LOXs. Inhibition of ACSL4, LPCAT3, and LOXs prevents lipid peroxidation and prevents ferroptosis.

### 2.3 Iron Metabolism

Iron's dual role in essential biological functions and ferroptosis cell death makes it a promising therapeutic target for treating both cancer and organ injuries. The biological significance of iron transcends its canonical functions, with disrupted iron metabolism now recognized as a central player in numerous pathological processes. Most notably, ferroptosis is a unique type of programmed cell death characterized by iron‐catalyzed accumulation of lipid peroxides, has garnered significant attention. This pathway's therapeutic potential, particularly in cancer treatment and organ protection strategies, exemplifies the complex duality of iron as both an indispensable cellular constituent and a potential mediator of oxidative injury [40]. Iron homeostasis is maintained through tightly regulated absorption and metabolic processes in healthy conditions; Fe^3+^binds to transferrin (TF) and enters the cell via the membrane protein TF receptor (TFR1). This pathway ensures the controlled delivery of iron to cells, enabling its utilization in essential biochemical processes. Once internalized, the iron–TF complex is trafficked to endosomes, where Fe^3+^ is released in an acidic environment. Then, six transmembrane epithelial antigen of prostate 3 acts as a metalloreductase to convert ferric iron (Fe^3+^) to ferrous iron (Fe^2+^), which is then transported by (DMT1) Divalent Metal Transporter 1 into labile iron pools [41]. These versatile iron repositories occupy a pivotal position in cellular iron homeostasis, orchestrating a delicate balance between serving as accessible metabolic resources and potentially triggering oxidative cascades when regulatory mechanisms are compromised. Excess iron catalyzes the Fenton reaction, generating hydroxyl radicals that initiate lipid peroxidation of membrane PUFAs, ultimately leading to ferroptosis [42, 43]. The presence of Fe^2+^ aggravates oxidative stress in cells, leading to ferroptosis. Research shows that modulating iron metabolism can disrupt lipid oxidation balance in cancer cells, making iron‐targeted therapies a promising approach for cancer treatment. By limiting iron‐dependent lipid peroxidation or enhancing ferroptosis in cancer cells, these strategies hold promise for developing novel anticancer therapies [44].

### 2.4 Autophagy

Recent investigations have uncovered that autophagy, a vital process for cellular homeostatic maintenance and metabolic equilibrium, exhibits substantial crosstalk with ferroptosis. This emerging understanding reveals the complex relationship between these two essential cellular processes. In both fibroblasts and pathological cells, the autophagic breakdown of ferritin can trigger ferroptosis. This observation suggests that under specific conditions, ferroptosis could be categorized as a type of cell death that relies on autophagy pathways. The ferroptosis inducer erastin is known to stimulate excessive autophagic activity, leading to enhanced ferroptosis through the accumulation of iron and oxidation of lipids. Studies have demonstrated that in cancer cells, the autophagic process plays a vital role in facilitating erastin‐mediated ferroptosis [45, 46, 47]. Nuclear receptor coactivator 4 (NCOA4) serves as a critical mediator of ferritinophagy, an optional autophagy‐mediated process specifically targeting ferritin degradation. Through its selective binding to ferritin and subsequent recruitment to autophagosomes, NCOA4 facilitates lysosomal degradation, giving rise to amplified levels of free intracellular iron. This elevation in free iron sensitizes cells to ferroptosis pathways. The mechanistic relationship between NCOA4‐mediated ferritinophagy and iron homeostasis not only reveals sophisticated regulatory networks linking autophagy to iron metabolism but also suggests promising therapeutic strategies. Specifically, modulation of NCOA4‐dependent ferritinophagy may offer novel therapeutic approaches for diseases characterized by iron dysregulation, including various malignancies and neurodegenerative disorders [48, 49]. The relationship between autophagy and ferroptosis is critically mediated by ROS‐dependent mechanisms. As cellular signaling molecules, ROS initiate autophagic responses under stress conditions. This process facilitates ferritin degradation through autophagy, resulting in expanded intracellular labile iron pools that amplify oxidative stress via enhanced lipid peroxidation, thereby promoting ferroptosis [50, 51]. ATG5 and ATG7 are critical factors in the initiation and progression of autophagy [52]. These autophagy‐related proteins are crucial for the formation of autophagosomes, the double‐membraned structures responsible for sequestering cellular components targeted for degradation. In cellular models deficient in ATG5 or ATG7, the disruption of autophagy impairs ferritin degradation, resulting in a significant reduction in intracellular labile iron levels. By lowering free iron levels, both ROS production and lipid peroxidation are diminished, leading to ferroptosis prevention [53]. The intricate interplay between autophagy and ferroptosis offers promising avenues for developing novel therapeutic strategies targeting diseases correlated with dysregulated iron metabolism pathways.

### 2.5 Reactive Oxygen Species

ROS function as essential mediators during ferroptosis. As intrinsically reactive oxygen derivatives, ROS orchestrate vital cellular signaling cascades and maintain homeostatic equilibrium under physiological conditions. Nonetheless, when ROS accumulate excessively, they cause severe oxidative damage to cellular components like lipids, proteins, and DNA, which can lead to multiple forms of cell death, particularly ferroptosis. As an iron‐dependent form of programmed cell death, ferroptosis features the accumulation of peroxidized lipids, catalyzed mainly by ROS [54]. This distinctive mode of cellular demise stands apart from conventional death pathways such as apoptosis, necrosis, and autophagy, characterized fundamentally by its signature process. Lipid peroxidation which depends critically on ROS‐mediated oxidative assault on membrane‐bound PUFAs. The progressive accumulation of lipid peroxides simultaneously compromises membrane structural integrity and initiates lethal signaling cascades. The three canonical metabolic pathways implicated in ferroptosis converge on ROS generation, ultimately culminating lipid peroxidation and the activation of ferroptosis. These interconnected metabolic networks encompass iron homeostasis, GSH biosynthesis, and lipid regulation. Perturbations in iron equilibrium elevate bioavailable redox‐active iron, while concurrent GSH depletion and GPX4 inactivation compromise cellular capacity to neutralize lipid peroxides. This metabolic convergence generates a profound prooxidant environment, accelerating ROS generation and lipid peroxidation, ultimately establishing conditions conducive to ferroptosis. Specifically, iron catalyzes the Fenton reaction, producing large amounts of potent hydroxyl radicals. The formed radicals catalyze PUFA peroxidation, generating accumulated lipid ROS. A decrease in GPX4 levels results in a rapid accumulation of lipid ROS within cells, ultimately triggering ferroptosis [55, 56, 57]. In light of these mechanisms, it is evident that ROS play a central and indispensable role in the activation and propagation of ferroptosis.

## 3 Ferroptosis and TIME

As immunotherapy continues to evolve, a more comprehensive understanding of the TIME is unveiling a myriad of potential therapeutic targets. Extensive research has elucidated the sophisticated interrelation between tumor cells and immune cells within this complex milieu. Ferroptosis activation not only restricts tumor growth but also shows promise in enhancing immunotherapeutic responses and overcoming resistance to conventional cancer treatments [58, 59]. However, it is crucial to note that targeted ferroptosis‐inducing therapies may potentially lead to the development of new resistance mechanisms. Ferroptosis significantly modulates immune cell function within the TIME. Conversely, In the TIME, immune cells boost cancer defense by triggering ferroptosis, establishing a bidirectional relationship. This dynamic interplay not only influences tumor growth and progression but also opens up novel avenues for cancer therapeutics [60, 61, 62, 63, 64]. Throughout tumor development, growth factors and chemokines secreted by neoplastic cells dramatically alter the composition of the TIME. The proportion and phenotypic characteristics of T cells emerge as critical determinants of tumor progression [13]. Multiple types of immune cells—macrophages, regulatory T (Treg) cells, DCs, and NK cells—coordinate antitumor immunity. Furthermore, stromal components, particularly cancer‐associated fibroblasts (CAFs), support tumor advancement by interacting with both immune and tumor cells [65, 66, 67]. It can be seen that tumor and immune microenvironment interact with each other and affect tumor progression.

**FIGURE 2:**
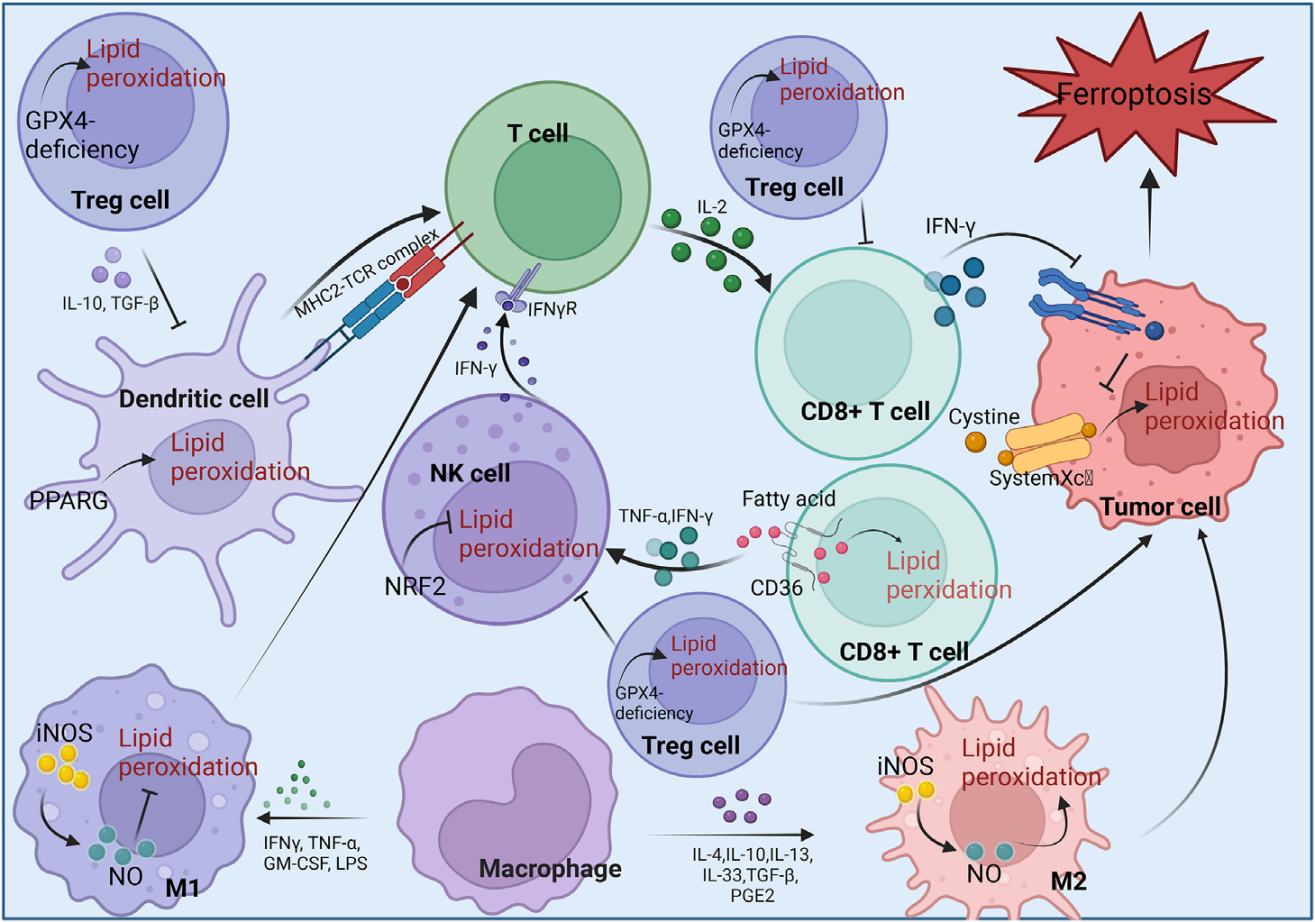
Crosstalk regulation of ferroptosis and immune cells in tumor microenvironment. The secretion of interferon (IFN‐γ) by CD8+ T cells affects ferroptosis in tumor cells, and the uptake of CD36 by CD8+ T cells results in ferroptosis. Compared with M1 macrophages, M2 macrophages lack iNOS and NO and are more prone to ferroptosis. Lipid peroxidation of Treg cells, NK cells and DCs causes ferroptosis.

Accumulating evidence demonstrates that the dynamic interplay between tumors and their immune microenvironment profoundly influences tumor progression and evolution. Recent investigations have unveiled intricate relationships between ferroptosis and immune cells within the TME [68, 69]. Recent studies have explored how ferroptosis connects with immune cells in TIME, such as the relationship between CD8+ T cells, macrophages, Treg cells, DCs, NK cells, and ferroptosis, and using ferroptosis as an immunotherapy target for cancer treatment has also been discussed [70, 71] (Table 1 and Figure 2). Interactions between tumor cells and immune cells in the microenvironment affect the immune response to subsequent immunotherapy.

**TABLE 1:** Role and regulatory mechanism of ferroptosis in immune cells.

Cell type	Target point	Effect	Induced changes in the immune microenvironment	References
CD8+ T cells	SLC7A11, SLC3A2↓	Secretion of various cytokines, such as IFN‐γ, and plays a role in immunity	Tumor killing	[72, 73]
	ACSL4↑		Tumor killing	[74, 75]
	CD36↑		Kills CD8+ T cells	[76, 77]
Macrophage	iNOS, NO↑	Antigen presentation, participates in immune response	M1 proinflammatory, M2 anti‐inflammatory	[78, 79]
	APOC1↓		Transformation of M2 to M1 macrophages, tumor killing	[80]
Treg cell	GPX4↓	Antigen tolerance to tumor cells is generated, and immune escape occurs in tumor cells	Tumor killing	[81]
NK cell	NRF2	Enhanced killing of tumors and viruses	Tumor killing	[82]
DC	PPARG	Antigen presentation	Tumor promoting	[83]

### 3.1 CD8+ T Cells and Ferroptosis

Immune cells play a large role in the occurrence of ferroptosis. As key regulators of the TIME and immune responses, immune cells not only modulate ferroptosis in tumor cells but are also themselves susceptible to ferroptosis under certain conditions. This dual role highlights the complicated interaction between ferroptosis and immune‐mediated processes in cancer progression and therapy. Current studies have indicated that interferon (IFN‐γ) secreted by CD8+ T cells downregulates SLC7A11 and SLC3A2 on Xc−, resulting in lipid peroxidation, which causes ferroptosis in tumor cells [72, 73]. The (SLC3A2) Solute Carrier Family 3 Member 2 protein on Xc− maintains SLC7A11 stability, and the inhibition of SLC7A11 can promote lipid peroxidation. Ferroptosis induction is modulated by the coordinated function of SLC7A11 and SLC3A2. In addition, IFN‐γ can interact with arachidonic acid, and the activation of ACSL4 can cause ferroptosis in tumor cells [74, 75]. Ferroptosis not only affects the TIME but also has a certain effect in promoting the immunosuppressive TME. Some studies have indicated that CD36 can damage CD8+ T cells and affect the antitumor effect of CD8+ T cells. CD36, a critical lipid transport receptor highly enriched in tumor‐associated CD8+ T cells, mediates the selective uptake of oxidized lipid moieties. This molecular mechanism simultaneously deteriorates CD8+ T cell metabolic fitness and promotes ferroptosis, consequently weakening their tumor‐combating potential. In the immunosuppressive TME, CD36‐mediated fatty acid uptake triggers lipid peroxidation and subsequent ferroptosis in CD8+ T cells [76, 77]. The antitumor effect of CD8+ T cells is impaired. Strategic interventions targeting CD36 functionality or fortifying CD8+ T cell resistance to ferroptosis present promising therapeutic opportunities for enhancing antitumor immune responses and dismantling the immunosuppressive architecture of the TME.

### 3.2 Macrophages and Ferroptosis

Macrophages and ferroptosis interact with each other, but the mechanism of action between ferroptosis and the TME is unclear [84]. Macrophages are vital immune cells. The effects of M1 and M2 macrophages are different, and their sensitivity to iron is also different. M1 macrophages have proinflammatory effects that limit tumor progression and kill tumor cells. M2‐polarized macrophages, characterized by their relatively low iron content, exhibit a multifaceted functional profile; they demonstrate potent anti‐inflammatory and immunosuppressive properties [85]. Induced nitric oxide synthase (iNOS) and nitric oxide (NO) free radicals are present in M1‐type macrophages, and iNOS and NO limit lipid peroxidation [78]. In contrast, M2 macrophages exhibit enhanced ferroptosis sensitivity relative to their M1 counterparts [79]. Studies show that blocking APOC1 promotes M2‐to‐M1 macrophage reprogramming via ferroptotic pathways. This phenotypic shift in macrophage polarization has demonstrated promising therapeutic potential in the context of hepatocellular carcinoma (HCC) treatment [80]. The interplay between macrophages and ferroptosis has been shown to significantly influence cancer treatment outcomes. Future research will employ experimental analyses to investigate the potential therapeutic effects of harnessing both ferroptosis and macrophage activity in the therapy of lung cancer and other pulmonary diseases.

### 3.3 Treg Cells With Ferroptosis

In the TME, Treg cells produce antigenic tolerance to tumor cells, which causes the immune escape of tumor cells, and targeting these cells is very important for enhancing antitumor immunity. Treg cells, a CD4+ T cell subpopulation, help preserve immune homeostasis through their immunosuppressive functions. However, in the TME, these cells are co‐opted to support tumor growth by suppressing the function of effector T cells and diverse immune populations that would otherwise attack tumor cells. This immunosuppressive function creates an environment conducive to tumor progression and immune evasion, making Treg cells a significant barrier to effective antitumor immunity [86]. Treg cells can facilitate tumor cell proliferation. By suppressing the activity of cytotoxic CD8+ T cells, NK cells, and other components of the immune system, Treg cells actively facilitate tumor immune escape. Through secretion of growth factors and cytokines, Treg cells both dampen immune responses and facilitate tumor progression, angiogenesis, and metastasis [87]. This dual role in immunosuppression and tumor promotion underscores the critical influence of Treg cells in shaping the TME. Research has demonstrated that GPX4 is critical in in mitigating lipid peroxidation and ferroptosis in Treg cells. Consequently, the downregulation of GPX4 can induce ferroptosis in Treg cells, potentially giving rise to enhanced tumor growth inhibition [81]. Killing tumor cells by inducing ferroptosis through the lipid peroxidation of Treg cells is an effective treatment strategy. The strategic induction of ferroptosis in Treg cells effectively diminishes immunosuppression within the TME, unleashing the full potential of cytotoxic immune cells to recognize and destroy cancer cells. Significantly, this ferroptosis‐driven Treg depletion approach shows remarkable potential for synergistic combination with existing immunotherapies, particularly checkpoint inhibition, amplifying their therapeutic efficacy. As research continues to illuminate the complex relationships between ferroptosis mechanisms and immune regulation, the targeted modulation of GPX4 and lipid peroxidation pathways in Treg cells represents a promising frontier in developing next‐generation cancer therapies that harness both ferroptosis and immunomodulatory mechanisms.

### 3.4 NK Cells and Ferroptosis

NK cells kill tumor cells and viruses, maintain the body's immune function, and have powerful antitumor effects [88]. Some studies have indicated that NK cells are associated with ferroptosis, and lipid peroxidation cause damage to the function of NK cells in the TME [82]. Additionally, the NRF2 (nuclear factor erythroid 2‐related factor 2) antioxidant pathway can restrain the functional damage of NK cells caused by ferroptosis and further strengthen the antitumor effect [82]. The relationship between NK cells and ferroptosis in the TME demands deeper study. The study has revealed the detrimental impact of ferroptosis on NK cell function and established the critical protective role of the NRF2 pathway, though the precise molecular mechanisms underlying these interactions await full characterization. Understanding the complex relationships between ferroptosis and NK cell metabolism, signaling cascades, and cytotoxic functions could uncover innovative strategies for optimizing NK cell performance in the TME. Furthermore, developing approaches to optionally trigger ferroptosis in tumor cells while protecting NK cells could yield powerful therapeutic synergies. This promising research direction may significantly advance cancer immunotherapy by strategically leveraging the interplay between ferroptosis and NK cell‐mediated immune responses.

### 3.5 DCs and Ferroptosis

DCs are effective antigen‐presenting cells in the body and maintain the differentiation and homeostasis of Treg cells. As key orchestrators of immune responses, DCs perform multiple critical functions: they survey the environment, process antigenic material, and display antigens to T lymphocytes, thus coordinating both immediate and learned immune responses. Additionally, DCs play a vital role in sustaining Treg cell development and stability, helping maintain the equilibrium between immune stimulation and regulation. Studies have indicated that there is a connection between DC dysfunction and lipid accumulation in cancer [89, 90, 91]. In the TME, ferroptosis can hinder the function of DC, and lipid oxidation has a blocking effect on antigen presentation in DCs. Ferroptosis has a negative effect on antigen presentation and may affect the efficacy of cancer immunotherapy [92, 93]. In another study, it was found that PPARG‐mediated ferroptosis hindered the maturation of DCs and their antitumor function. (PPARG) Peroxisome Proliferator‐Activated Receptor Gamma, a nuclear receptor known for its role in lipid metabolism, contributes to the induction of ferroptosis in DCs. This process disrupts the functional integrity of DCs and diminishes their ability to activate T cells, thereby weakening the antitumor immune response. This finding underscores the critical link between lipid metabolism, ferroptosis, and DC dysfunction in the TME [83]. These studies indicated that ferroptosis has a negative function on DCs in the TME and that restraining ferroptosis can enhance the immune response of DCs. The strategic inhibition of ferroptosis in DCs presents a compelling therapeutic approach for enhancing their antigen‐presenting capabilities and strengthening antitumor immune responses. Through preservation of DC maturation and functional integrity, ferroptosis suppression may potentiate existing immunotherapeutic modalities. Moreover, elucidating the molecular mechanisms underlying ferroptosis‐mediated DC dysfunction could facilitate the development of precision therapeutics designed to restore DC functionality and augment immune responses within the TIME. These observations underscore the fundamental importance of continued investigation into the relationship between ferroptosis processes and DC biology in cancer progression.

### 3.6 Other Components of the Microenvironment and Ferroptosis

While immune cells play a pivotal role in ferroptosis within the tumor immune microenvironment, other components of this complex milieu also exert significant influence on this process. Notably, CAFs are now recognized as important determinants of ferroptosis sensitivity [94, 95, 96]. Research has revealed that CAFs secrete cysteine, which contributes to maintaining cellular redox homeostasis, thereby enhancing resistance to ferroptosis in tumor cells [97]. CAFs have been indicated to suppress the cytotoxic activity of NK cells, thereby promoting tumor immune evasion. Interestingly, under conditions of iron depletion, ferroptosis is attenuated, which in turn protects NK cells from CAF‐induced ferroptosis. This iron‐dependent mechanism highlights the complex interrelation between stromal cells, immune effectors, and metabolic processes in the TME [98]. Evidently, the complicated crosstalk between the TME and ferroptosis significantly impacts tumor progression. The modulation of cysteine metabolism by CAFs emerges as a promising avenue for further investigation in the treatment of lung cancer and other pulmonary diseases. This metabolic reprogramming represents a potential therapeutic target, offering new insights into strategies for combating these challenging malignancies.

## 4 Ferroptosis in Pulmonary Diseases

**FIGURE 3:**
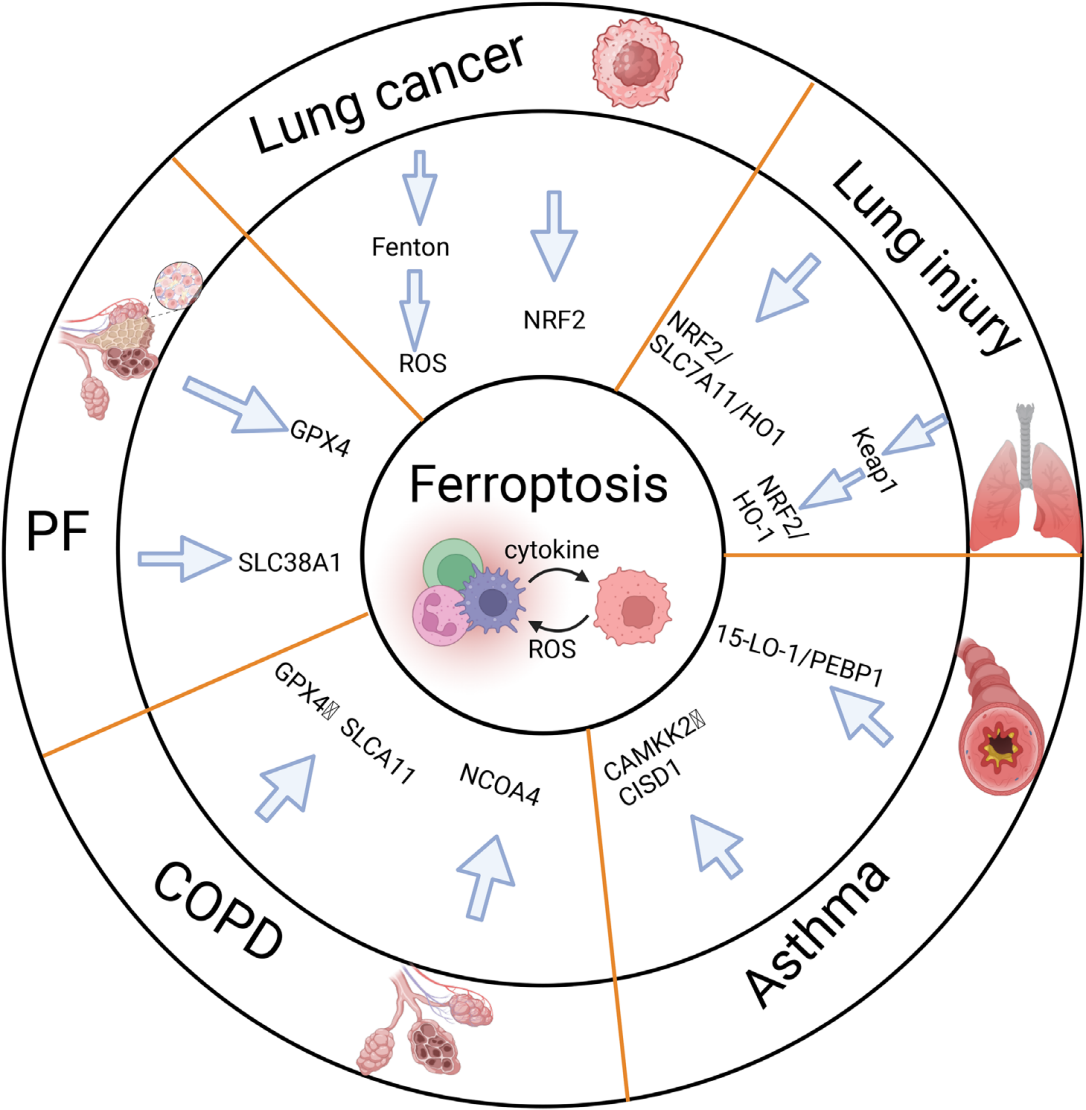
Overview of ferroptosis, lung cancer and pulmonary disease. Ferroptosis is a new target for treating cancer. Ferroptosis is triggered by lipid peroxidation and iron accumulation. Many ferroptosis regulators, such as GPX4 and NRF2, are key players in ferroptosis.

**FIGURE 4:**
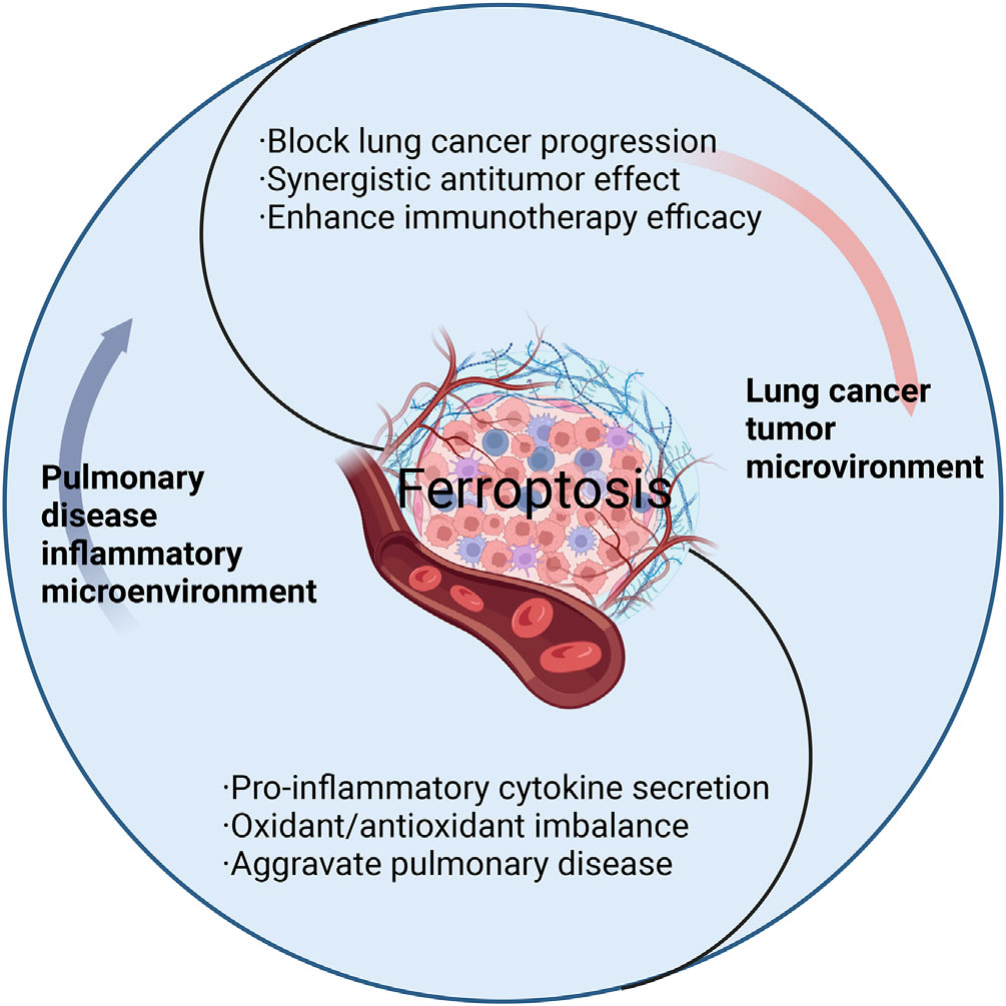
Role of ferroptosis in immune microenvironment of lung cancer and pulmonary disease. The connections between ferroptosis in the TME and the inflammatory microenvironment are different. The effects on patients are also different, the inflammatory microenvironment can bring about proinflammatory cytokine secretion, oxidant/antioxidant imbalance, and aggravate pulmonary disease. The tumor microenvironment leads to block lung cancer progression, synergistic antitumor effect, and enhances immunotherapy efficacy.

**FIGURE 5:**
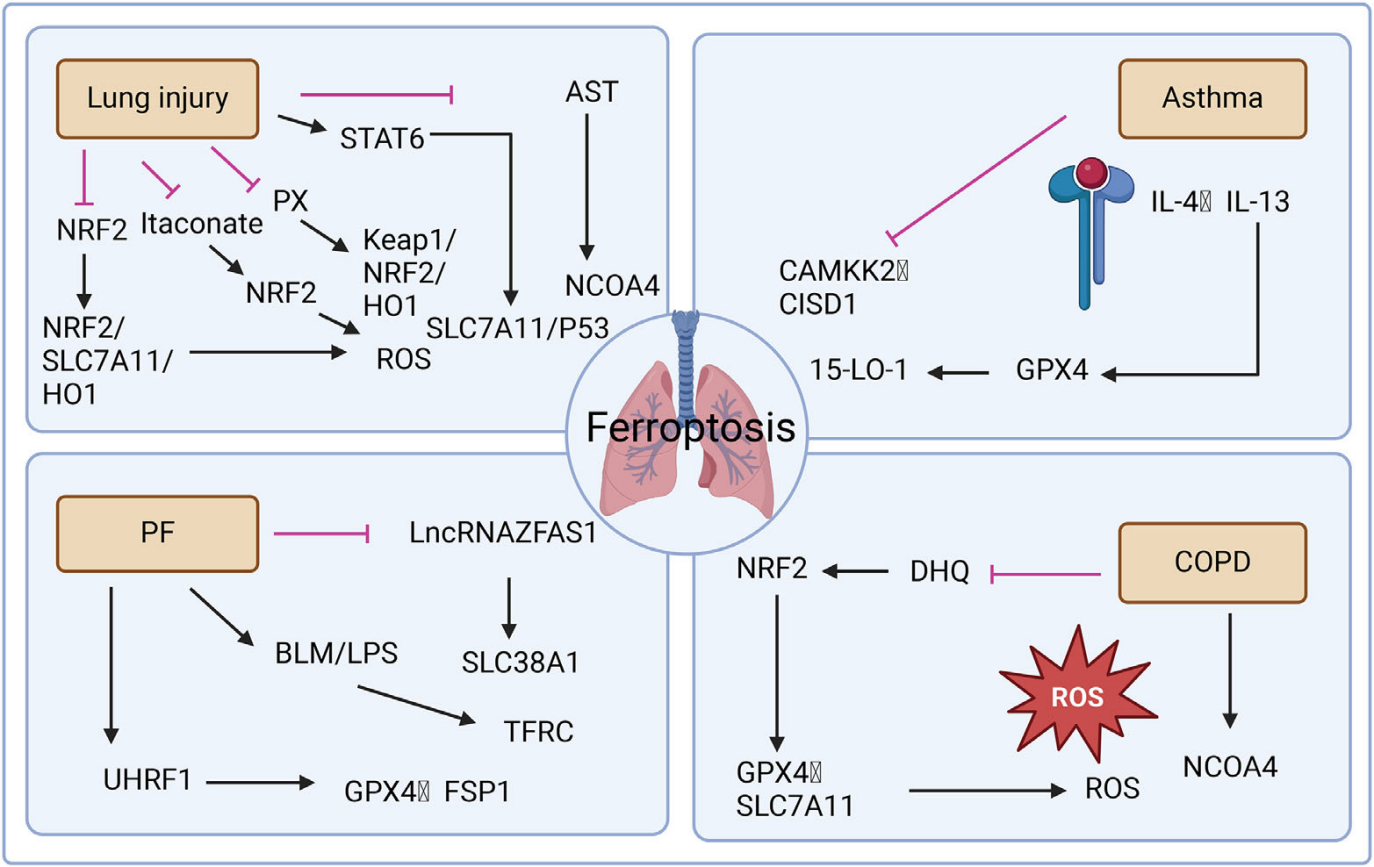
Schematic of the mechanism of ferroptosis in pulmonary diseases. The regulatory factors of ferroptosis in pulmonary diseases (lung injury, asthma, COPD, PF) are different, but various pulmonary diseases are associated with the accumulation of ROS, which causes redox imbalance and ferroptosis.

Targeting ferroptosis is regarded as a new method for treating cancer. At present, numerous studies have not only made great breakthroughs in the study of ferroptosis in the therapy of cancer [99, 100, 101], but also have numerous discussions in the therapy of other diseases, such as lung injury, pulmonary fibrosis (PF), asthma, chronic obstructive pulmonary disease (COPD), and other pulmonary diseases [102, 103, 104, 105] (Figure 3). Furthermore, the impact of ferroptosis varies significantly between TMEs and inflammatory microenvironments, highlighting its dual role in disease treatment. In the context of TMEs, ferroptosis acts as a potent inhibitor of lung cancer progression, synergizing with antitumor mechanisms and potentially enhancing the efficacy of immunotherapies. Conversely, within inflammatory microenvironments associated with various pulmonary diseases, ferroptosis can exacerbate pathological conditions by promoting the generation of inflammation‐inducing signals and disrupting the delicate balance between oxidative and antioxidative processes, thereby aggravating the onset and progress of pulmonary diseases (Figure 4). We discussed how associations between ferroptosis and immune microenvironment contributes to pulmonary disease (Figure 5).

### 4.1 Lung Injury

(ALI) Acute Lung Injury occurs when lung epithelial and capillary endothelial cells are damaged through direct or indirect mechanisms. The pathogenesis of ALI involves oxidative stress, which affects the progression of ALI. Ferroptosis is a regulated cell death process that requires iron, is triggered by the accumulation of lipid peroxides and ROS, which are closely linked to the oxidative stress mechanisms in ALI. Regulatory factors such as GPX4, SLC7A11, and NRF2 are critical in controlling ferroptosis and significantly impact determining the severity of lung injury. Dysregulation of these factors exacerbates ferroptosis, contributing to the progression of ALI, ALI is affected by various regulatory factors involved in ferroptosis [106, 107, 108, 109]. In ALI, studies have shown that lung injury can be alleviated through the NRF2/SLC7A11/HO1 pathway. The NRF2/SLC7A11/HO‐1 pathway is a vital antioxidant defense mechanism that protects cells from ferroptosis and oxidative stress‐induced injury. NRF2, a transcription factor, is crucial for regulating cellular redox homeostasis by inducing the expression of antioxidant genes, including SLC7A11 and HO‐1, NRF2 inhibits ferroptosis via SLC7A11 and HO‐1, and when NRF2 gene expression is reduced, lipid peroxidation occurs, thus aggravating lung injury [106, 107, 108, 109, 110]. Ferroptosis exacerbates lung injury, while ferroptosis inhibitors and lipid peroxidation inhibitors can alleviate lung injury. For example, Astaxanthin, a potent antioxidant derived from marine organisms, stabilizes intracellular iron levels by upregulating ferritin, an iron storage protein, and downregulating NCOA4, a key mediator of ferritinophagy. This dual action prevents the accumulation of free iron and lipid peroxides, thereby protecting lung cells from ferroptosis‐induced damage [111, 112]. Panaxydol (PX), a bioactive compound found in ginseng, has been indicated to exert protective function against lung injury by activating the Keap1–Nrf2/HO‐1 pathway. By inhibiting Keap1, an Nrf2 suppressor, PX promotes Nrf2 activation and upregulates HO‐1 expression, enhancing cellular resistance to ferroptosis and oxidative stress. This emphasizes the therapeutic potential of natural compounds in modulating ferroptosis and alleviating ALI [113]. Another study indicated that the expression of STAT6, a regulator of ferroptosis, was positively correlated with that of SLC7A11. By upregulating SLC7A11, STAT6 enhances GSH synthesis and reduces lipid peroxidation, thereby restraining ferroptosis. Furthermore, STAT6 suppresses P53 activity, a key proferroptotic factor, through posttranslational modifications, mitigating its sensitizing effects on ferroptosis. p53 is also a key factor in ferroptosis and sensitizes cells to ferroptosis by downregulating SLC7A11. That is, SLC7A11/P53 can reduce the harm caused by lung injury [114, 115, 116]. At present, immunotherapy is widely used, and the interaction between the TME and ferroptosis affects the efficacy of lung injury treatment. Itaconate can decrease the production of ROS and the formation of lipid peroxides through the NRF2 pathway, thereby inhibiting ferroptosis in macrophages and thus alleviating sepsis‐induced lung injury. The interaction between immune cells and ferroptosis in the TME further affects lung injury [117]. Targeting the relevance between the TME and ferroptosis may be more conducive to alleviating acute lung injury. By addressing the crosstalk between ferroptosis, immune cells, and the TME, therapeutic strategies can be developed to reduce oxidative stress, enhance immune function, and protect lung tissues, offering new hope for the treatment of ALI.

### 4.2 Asthma

Ferroptosis is strongly linked to asthma. Inhibiting ferroptosis can alleviate airway inflammation in patients with asthma, and the occurrence and progression of asthma involves the immune system. In asthma, the primary manifestation is blocked airways, involving both immune cell invasion and activation, while the ongoing communication between immune and epithelial cells advances the disease process [118, 119]. A chronic airways disorder, asthma features inflammatory processes marked by oxidative stress and disrupted immune responses. Asthma's inflammatory response and airway epithelial cell damage have been connected to ferroptosis, a specific type of programmed cell death that depends on iron and involves lipid peroxidation [120, 121]. Recent investigations have revealed a significant correlation between house dust mite‐induced asthma and increased lipid peroxidation and ROS production. The research indicates a possible underlying connection between how asthma develops and ferroptosis processes [122]. Based on previous research, the use of ferroptosis inhibitors helps preserve the functional activity of both GPX4 and SLC7A11, thereby preventing ferroptosis. The increased activity of 15‐LO‐1 in cells is an important discovery in asthma research [123, 124]. LOX is the core participant of ferroptosis, which arises from the accumulation of PUFAs, and ferroptosis is driven by the peroxidation of PUFAs by LOX [125, 126]. Studies on LOX have shown that activation of 15‐oxygenase‐1 (15‐LO‐1) of IL13/IL4 and high expression of phosphatidylethanolamine‐binding protein 1 (PEBP1) in T2 inflammation can intensify the progress of asthma. Therefore, inhibiting the expression of 15‐LO‐1 and PEBP1 can reduce asthma [127]. Moreover, IL‐4 and IL‐13 restrain the expression of GPX4 and increase the level of 15‐LO‐1 in patients with asthma, and a decrease in GPX4 activity exacerbates the occurrence of asthma. In patients with asthma, genes associated with ferroptosis can influence the immune microenvironment. The study has indicated that upregulated CISD1 and CAMKK2 can restrain ferroptosis and alleviate the occurrence of asthma. The membrane protein CISD1 contains iron, while CAMKK2 belongs to the serine/threonine protein kinase group—both having significance in asthma pathways. This study revealed that CAMKK2 and CISD1 are inhibitors of ferroptosis [128]. CISD1 expression and Treg cells were found to be negatively correlated. CISD1 is linked to the microenvironment, and another study revealed that asthma can be alleviated by inhibiting Treg cells in mice [129]. However, Further investigation is necessary to determine how ferroptosis and the immune microenvironment are connected in asthmatic conditions.

### 4.3 Chronic Obstructive Pulmonary Disease

COPD is a widespread chronic respiratory condition affecting the airways. There are many causes of COPD, among which smoking is the most common [130]. The development of COPD is linked to ferroptosis. Mouse models exposed to CS demonstrated increased iron and lipid peroxide levels in lung epithelial cells, activating ferroptosis. Studies indicate that controlling ferroptosis could offer a therapeutic approach for COPD [131, 132]. A study has indicated that dihydroquercetin (DHQ) restrains ferroptosis in smoking‐induced COPD by activating NRF2. Treatment with DHQ inhibits ferroptosis by enhancing GPX4 and SLC7A11 expression while reducing lipid peroxide formation in cells [133, 134]. Currently, immunotherapy is becoming more developed and more widely applied to treat all kinds of diseases. Studies have indicated that targeting ferroptosis and immune cells in the TME can be used to treat COPD in both COPD patients and CS‐exposed mice. NCOA4 can mediate ferroptosis by polarizing M2‐type macrophages, and NCOA4 overexpression can accelerate ferroptosis. Moreover, NCOA4 can maintain iron homeostasis in vivo during ferroptosis via ferritin autophagy [53, 135, 136]. Targeting NCOA4 through inhibition could offer a new direction in COPD therapy.

### 4.4 Pulmonary Fibrosis

PF is also referred to as interstitial pulmonary disease. PF is a condition in which normal tissue is replaced by fibrous tissue, resulting in lung damage [137, 138]. There are many factors that cause PF, such as environmental factors and ionizing radiation [139], and PF is irreversible and can only be alleviated by some therapeutic methods. With the discovery of ferroptosis, ferroptosis has become a concern in various diseases. The role of ferroptosis in PF has become a growing area of scientific interest, offering new therapeutic possibilities [140]. Moreover, immune cells in the TME have connections with PF, such as DC [141]. In the study of pulmonary fibrosis, it was found that the de novo methylation of the UHRF1 gene can increase the methylation of the GPX4 and FSP1 genes, thus suppressing gene expression and subsequently inducing ferroptosis, promoting the occurrence of PF. Targeting UHRF1 may be an effective treatment strategy for PF [142]. Studies have shown that bleomycin (BLM) and lipopolysaccharide (LPS) are inducers of PF, and under the induction of BLM or LPS, transforming growth factor‐β (TGF‐β) can regulate the expression of TF receptor protein 1 (TFRC) in lung fibroblasts, resulting in increased iron accumulation in cells and further leading to ferroptosis. Ferroptosis inhibitors can inhibit PF in lung epithelial cells induced by BLM or LPS; that is, inhibiting ferroptosis can alleviate PF [143]. PF was also found to be mitigated by the downregulation of SLC38A1, a key factor of lipid peroxidation. The lncRNA zinc finger antisense 1 (lncRNAZFAS1) expression was enhanced in rat lung tissue with BLM‐induced PF and in HFL1 cells exposed to TGF‐β1. This upregulation showed positive correlation with SLC38A1 and led to ferroptosis [144]. Therefore, the inhibition of lncRNAZFAS1 can alleviate the progress of PF.

Numerous studies have indicated that inhibiting ferroptosis relieves PF. Nanotechnology has been increasingly used to treat diseases. It is found that ferroptosis inhibitors are difficult to accurately reach the target position, and nano‐drugs have good biocompatibility, and relevant nanomedicine are designed through the action mechanism of ferroptosis, and then realize the diagnosis and progress of multifunctional nanomedicine diseases based on ferroptosis [145, 146, 147, 148, 149]. And some studies have indicated that liposomes are the most potential nanomedicine, and it is a good direction to treat PF through nanotechnology [150]. However, there are still many problems to be broken through in the progress of the combined implementation of nano‐scale technologies and ferroptosis in the treatment of diseases, such as clinical treatment and toxicity after superimposed use, and further research on the combined treatment based on ferroptosis [151]. At present, the mechanism of ferroptosis and immune microenvironment on PF is not clear (Table 2).

**TABLE 2:** Therapeutic targets of ferroptosis in pulmonary disease.

Pulmonary disease	Target/agent of action	Signaling pathway/correlation factor	Causes changes in pulmonary disease	References
Lung injury	NRF2	NRF2/SLC7A11/HO1	Remission	[110]
	AST	NCOA4		[111, 112]
	PX	Keap1–Nrf2/HO‐1		[113]
	STAT6↑	SLCA11/P53		[114, 115, 116]
	Itaconate	NRF2		[117]
Asthma	15‐LO‐1, 16‐PEBP1↓	GPX4	Remission	[127]
	CISD1, CAMKK2↑	ROS		[128, 129]
COPD	DHQ	NRF2	Remission	[133, 134]
	NCOA4↓	Ferritin autophagy		[135, 136]
PF	UHRF1↓	GPX4/FSP1	Remission	[142]
	TFRC↓	Fe accumulation		[143]
	lncRNAZFAS1↓	SLC38A1		[144]

### 4.5 Other Pulmonary Diseases

In addition to the common pulmonary diseases (ALI, pulmonary fibrosis, asthma, and COPD). Ferroptosis is implicated in multiple respiratory diseases, with tuberculosis being one of the key examples. The occurrence of tuberculosis is related to mycobacterium tuberculosis (Mtb), and Mtb inhibits the function of macrophages [152, 153]. Studies have found a link between MTB‐induced macrophage death and GPX4 reduction, and there is also an increase in free iron and lipid peroxides. It has been found in mouse experiments that inhibiting ferroptosis can alleviate tuberculosis [154]. Staphylococcus aureus also causes a common pulmonary infection. Studies have shown that IFP35 can aggravate staphylococcus aureus infection by promoting ferroptosis. The macrophages of mice infected with staphylococcus aureus show an increase in IFP35, and IFP35 promotes the degradation of NRF2, leading to ferroptosis [155]. Targeting IFP35 could be an effective method to treat staphylococcus aureus infections. In addition, due to the occupational disease‐silicosis caused by silica, there are few studies on silicosis in the existing literature. Some studies have indicated that DHQ has been tested in vivo/in vitro. The study demonstrate that DHQ protects lung epithelial cells by enhancing GPX4 activity, which leads to decreased lipid peroxidation and subsequent reduction in ferroptosis [156]. The drug provides insights into the treatment of silicosis. HGF is very crucial in the prevention of fibrosis. TGF‐ β is a factor promoting fibrosis. Through the regulation of HGF and TGF‐ β, ferroptosis can be inhibited and PF can be alleviated [157]. However, it is still a great challenge to be used in clinical practice.

## 5 Ferroptosis in Lung Cancer

Lung cancer is also known as primary bronchial lung cancer. There are numerous factors that cause lung cancer, such as smoking, air pollution, and occupational hazards, among which smoking is currently considered the most dangerous factor. In recent years, ferroptosis has become a means of targeted cancer treatment [158, 159, 160, 161]. Research has demonstrated that inhibiting the ubiquitination of Heme Oxygenase 1 (HMOX1) and suppressing the activation of N‐alpha‐acetyltransferase 10 can effectively trigger ferroptosis in tumors. HMOX1 has emerged as a vital factor in the induction of ferroptosis, thus presenting a promising target for achieving therapeutic effects in various malignancies [162, 163]. Immunotherapy is widely used to treat cancer patients and greatly improves the survival rate of patients. Current studies have indicated that ferroptosis has become an important target for treating tumors, that immunotherapy is affected by ferroptosis, and that the interaction between the immune system and ferroptosis affects the treatment of cancer patients [164, 165]. Recent studies have revealed that targeting carnitine palmitoyltransferase 1A (CPT1A) can enhance lung cancer immunotherapy and potentially improve treatment outcomes. Research has identified that CPT1A, a crucial enzyme involved in fatty acid oxidation, enables lung cancer to resist ferroptosis while simultaneously suppressing CD8+ T cell activity. Two main mechanisms drive the CPT1A/c‐Myc positive feedback loop's enhancement of cellular antioxidant capacity: activation of the NRF2/GPX4 system and downregulation of ACSL4, effectively inhibiting ferroptosis and thereby augmenting the efficacy of lung cancer treatments [166, 167]. Current research exploring the intersection of ferroptosis and the immune microenvironment remains limited. The TME is an intricate ecosystem comprising various cell types, with its immune components collectively referred to as the immune microenvironment. Emerging evidence suggests that certain characteristics of ferroptosis may act as valuable indicators for clinical diagnosis and prognosis, demonstrating a notable association with the tumor immune microenvironment in lung cancer progression [168]. Moreover, in ongoing investigations into ferroptosis and the immune microenvironment, researchers have identified Circ_BBS9 as a novel early biomarker for lung cancer. This circular RNA interacts with IFIT3, playing a role in modulating the TIME [169].As described above, ferroptosis and immune cell generation in the TME can influence each other [170, 171, 172, 173, 174]. Studies have shown that nanotechnology can improve immunotherapy efficacy and reduce side effects [174, 175, 176]. Nanoparticles can be used to deliver iron to tumor cells. The path to ferroptosis in tumor cells occurs iron overload, which induces the Fenton reaction and drives the accumulation of lipid peroxides, eventually leading to cell death [177]. Ferroptosis in lung cancer cells can be triggered by NRF2 nanomodulators, while the TME can be activated through additional immunizations. NRF2 maintains cellular redox stability. In this study, zero‐valent iron nanoparticles (ZVI‐NPs) were shown to have dual anticancer effects. On the one hand, ZVI‐NPs can cause lipid peroxidation, which leads to ferroptosis. On the other hand, ZVI‐NPs can transform M2‐type macrophages into M1‐type macrophages, further strengthening their antitumor properties. This bifunctional nanomedicine can synergistically induce ferroptosis in cancer cells and is capable of reprogramming the immunosuppressive microenvironment [178]. The dual targeting of the microenvironment and ferroptosis is a promising therapeutic strategy for lung cancer treatment. New research has revealed Fe–TCPP–R848–PEG (Fe–MOF–RP), a light‐activated nanoenzyme capable of modifying the immunosuppressive microenvironment. This innovative material functions by promoting the conversion of M2 macrophages to M1 macrophages, subsequently inducing ferroptosis. Through this mechanism, Fe–MOF–RP further modifies the tumor immunosuppressive microenvironment [179]. The approach of designing nanoscale drugs for cancer treatment is gaining increasing attention in the research community. This emerging field of study opens up new avenues for cancer therapy, offering promising directions for future investigations. The continuous development and investigation of nanoengineered approaches is creating pathways toward more selective and efficient cancer treatments.

## 6 Other Cell Death Modalities in Lung Cancer and Pulmonary Diseases

Although current research on cell death in lung cancer and pulmonary diseases has primarily focused on ferroptosis, other cell death mechanisms also hold significant research value in this field. Further investigation into the mechanisms linking various forms of cell death to lung cancer and pulmonary diseases represents a promising research direction. This is not only because of the complex interaction networks that exist among different cell death pathways, but more importantly, existing studies have demonstrated that the synergistic relationship between autophagy and ferroptosis significantly influence the development and progression of lung cancer and pulmonary diseases.

### 6.1 Pulmonary Diseases

Recent studies have demonstrated that while ferroptosis maintains its prominent role in treating lung cancer and various pulmonary diseases, other cell death mechanisms have also garnered significant research attention. Ferroptosis, an autophagy‐dependent cell death process, can be enhanced through iron accumulation or lipid peroxidation during excessive autophagy [46]. Emerging research has revealed that empagliflozin (EMPA) targets both ferroptosis and autophagy, ameliorating BLM‐induced pulmonary fibrosis in rats through modulation of the Sesn2/AMPK/Nrf2 pathway. EMPA demonstrates therapeutic potential by attenuating oxidative stress, ferroptosis, and endoplasmic reticulum stress, while simultaneously enhancing Nrf2 expression, HO‐1 activity, and GPX4 levels to improve PF conditions [180]. The development of respiratory diseases has been strongly linked to PM2.5 exposure. The upregulation of HO‐1 expression represents a critical step in PM2.5‐induced ferroptosis and exacerbated lung fibrosis. HO‐1 facilitates hemoglobin decomposition and iron ion release in fibrotic cells, subsequently triggering mitochondrial ROS production and functional impairment. Furthermore, researchers have uncovered intricate connections between AMPK‐ULK1 axis‐activated autophagy, NCOA4‐mediated ferritinophagy, and PM2.5‐induced ferroptosis in fibrotic cells. PM2.5 exposure induces mitochondrial dysfunction and excessive ROS generation, promoting iron autophagy and intracellular iron accumulation, eventually culminating in ferroptosis of PF cells and disease progression [181]. Bisphenol A (BPA) has been indicated to promote oxidative stress and induce ferroptosis, thereby triggering lung fibrosis. The ferroptosis inhibitor Fer‐1 demonstrates partial mitigation of BPA‐induced effects. Mechanistically, BPA accelerates PF by activating the AMPK/mTOR signaling pathway and enhancing autophagy‐mediated ferroptosis, particularly affecting alveolar epithelial cells [181].

New studies demonstrate that Ficolin B, released by alveolar macrophage exosomes, worsens lung damage caused by BLM through ferroptosis activation via the cGAS–STING pathway. As a recognition molecule, Ficolin B exhibits diverse ligand‐binding capabilities and plays crucial roles in immune responses and homeostasis maintenance. Experimental studies demonstrate that macrophage‐derived Ficolin B aggravates BLM‐induced lung injury and fibrosis by promoting both autophagy and ferroptosis in lung epithelial cells through the cGAS–STING pathway [182]. NVP‐AUY922 exhibits immunomodulatory and antitumor properties. Research has validated its ability to block GPX4 pathway‐mediated autophagy both in vitro and in vivo. The autophagy inhibitor Baf‐A1 significantly elevates GPX4 levels and reduces lung inflammation. NVP‐AUY922 mitigates radiation‐induced lung injury by restraining GPX4 lysosomal degradation mediated by its partner protein, suggesting its potential as a novel therapeutic agent for radiation‐induced lung injury [183]. The study has demonstrated that paraquat disrupts mitochondrial homeostasis, increases AMPK phosphorylation, and activates the NCOA4–FTH axis. Notably, autophagy activation precedes ferritin degradation, and autophagy inhibition prevents ferritinophagy‐induced iron accumulation. The regulation of NCOA4‐mediated iron cycling and ferroptosis inhibition shows promise in ameliorating glyphosate‐induced lung injury [184].

The investigation of the interaction between ferroptosis and autophagy in pulmonary diseases has arisen as a promising research frontier, offering novel insights into the pathogenic mechanisms underlying complex respiratory disorders, including asthma and COPD. This expanding field of study has established a crucial theoretical framework for the development of more efficacious therapeutic interventions. As research in this domain continues to advance, it holds considerable promise for revolutionary breakthroughs in the precision medicine approach to pulmonary diseases.

### 6.2 Lung Cancer

Although cancer research has deeply examined ferroptosis mechanisms, the bidirectional communication between ferroptosis and autophagy processes requires more research attention. Studies have demonstrated the involvement of ferroptosis and autophagy in HCC, colorectal cancer, and lung cancer. Targeting the interplay between these cell death pathways may represent a promising therapeutic strategy for lung cancer treatment in the future. Although research on the interaction between ferroptosis and autophagy in lung cancer remains relatively limited, existing studies have demonstrated that combined suppression of autophagy and MEK signaling pathways can trigger ferroptosis in lung tumors characterized by LKB1 deficiency and KRAS activation [185]. Research indicates that in colorectal cancer, after ANXA10 deletion, this calcium‐dependent phospholipid‐binding protein (a member of the Annexin family) triggers ferroptosis by suppressing the autophagy‐dependent breakdown of TFRC [186]. Furthermore, research has shown that PNO1 restricts autophagy‐mediated ferroptosis by reprogramming GSH metabolism. As an inhibitor of ferroptosis, PNO1‐induced autophagy promotes the expression of the cystine/glutamate antiporter SLC7A11, leading to glutamate accumulation, enhancing system Xc− activity, and restricting the occurrence of ferroptosis. In the process of maintaining the redox homeostasis, PNO1 activates autophagy metabolism regulation to maintain the cysteine supply required for GSH synthesis, ultimately protecting HCC cells from ferroptosis through GSH metabolism reprogramming [187].

However, there is currently no systematic study on the crosstalk between autophagy and ferroptosis in lung cancer. Existing evidence indicates that autophagy and ferroptosis are essential in the occurrence, progression, and treatment resistance of lung cancer, but the synergistic mechanisms between the two and their potential impact on treatment remain unclear. Future investigations should emphasize elucidating the interconnected mechanisms between autophagy and ferroptosis processes, especially their potential value in targeted therapy and immunotherapy. In‐depth exploration in this area will provide innovative treatment approaches and combination treatment strategies for precision treatment of lung cancer, while promoting the translation of basic research findings into clinical applications.

## 7 Conclusion and Prospects

Ferroptosis is a novel iron‐dependent nonapoptotic cell death mode. Many studies have linked ferroptosis with cancer and neurological diseases [188, 189]. When the iron content in cancer cells increases, the Fenton reaction occurs, which causes ferroptosis; targeting ferroptosis may be useful for cancer treatment [190, 191, 192]. In addition, ferroptosis is regulated by many factors, such as GPX4, which is a key enzyme that prevents peroxidation. When GPX4 is inhibited, lipid peroxidation occurs, resulting in ferroptosis [193]. Despite advancements in cancer therapeutics, overcoming therapeutic resistance continues to be a major obstacle in cancer treatment. Consequently, a substantial body of preclinical and clinical studies is dedicated to elucidating mechanisms of drug resistance and developing strategies to overcome it. Emerging evidence suggests that ferroptosis, a form of regulated cell death, is intricately associated with cancer treatment resistance. Notably, inducing ferroptosis has demonstrated promise in reversing drug resistance in various cancer models [167, 194, 195]. Manipulating ferroptosis pathways may offer a promising therapeutic strategy for this condition.

The role of ferroptosis is complex, with both positive and negative consequences. Ferroptosis kills tumor cells in the TME but increases the severity of pulmonary disease by promoting the inflammatory microenvironment. The pathogenesis of ferroptosis is very important for the therapy of pulmonary disease and lung cancer. At present, the pathogenesis of ferroptosis includes GSH metabolism, iron metabolism and lipid metabolism. With the continuous progress of research, the pathogenesis of ferroptosis is more and more abundant. Ferroptosis is a very complex process in the immune microenvironment that involves interactions among CD8+ T cells, macrophages, Treg cells, DCs, NK cells, and ferroptosis [196]. Studies have shown that the combined effect of ferroptosis and immunotherapy shows a strong antitumor effect. For the treatment of diseases, moderate ferroptosis will increase the antitumor function, but excessive ferroptosis will affect the level of fatty acids. Further hindered the antitumor effect [197]. The application of immunotherapy combined with ferroptosis and immune microenvironment in clinical application is a major breakthrough for the future therapy of lung cancer and pulmonary diseases. The complex relationship between the two has influenced the development of immunotherapy, and the development and application of nanotechnology to generate drugs has increased. Studies investigating these drugs provide strong support for targeting ferroptosis to restrain tumor growth [198, 199, 200, 201, 202, 203, 204]. With the rapid development of science and technology, nanotechnology and nanomaterials continue to improve. Upon drug loading, nanomaterials disrupt cellular redox equilibrium through Fenton reactions, generating ROS that triggers ferroptosis‐mediated cell death [205, 206, 207]. Research demonstrates that besides Fenton reactions, ferroptosis can be induced by inhibiting GPX4, decreasing GSH content, and blocking System Xc− activity [151], as far as tumor tissue is concerned, the targeting capabilities of nanocarriers encompass both passive and active strategies. Compared with the normal tissue, the neovascularization of the tumor tissue is abundant, and the nano‐carrier can infiltrate into the tumor tissue through these loopholes. In addition, the lymphatic system in the tumor tissue is relatively lacking, so the clearance of the nano‐carrier is suppressed, these reasons lead to the passive targeting of nano‐carriers in tumor tissue. Synthesize nano‐vesicles to target tumor cells, further act on immune cells in TIME, activate the ferroptosis pathway, and further enhance the antitumor effect through synergism [208, 209, 210]. Research on the dual targeting of the immune microenvironment and ferroptosis by nanomedicine demonstrates the low‐toxicity and efficient antitumor effects of these drug systems. Nanomedicine represents a suitable direction for clinical research and has shown promise in improving the efficacy of treatment.

The intricate relationship between ferroptosis and TIME has positioned it as a key area of scientific research. This cell death mechanism exerts dual antitumor effects: directly eliminating tumor cells and modulating immune cells within the TIME, thereby enhancing antitumor immune responses. Future research endeavors will concentrate on elucidating the mechanisms underlying the connection between ferroptosis and the immune microenvironment. Key areas of investigation include exploring methods to augment immunotherapy efficacy through ferroptosis modulation and developing innovative strategies that synergistically combine ferroptosis inducers with immunotherapeutic approaches to increase the precision and effectiveness of cancer therapy. The integration of ferroptosis and immunotherapy demonstrates remarkable potential. This novel direction in cancer research not only offers promise in overcoming resistance to conventional therapies but also presents new avenues of hope for patients. In conclusion, this synergistic approach combining ferroptosis and immunotherapy may provide a new paradigm for cancer therapy, potentially improving patient outcomes and offering innovative solutions to current therapeutic challenges.

The recognition of ferroptosis has pioneered fresh directions in biomedical studies, revealing promising therapeutic potential across various diseases. Nevertheless, significant challenges remain that require systematic investigation and resolution. A primary concern in clinical applications is the potential cytotoxicity and adverse effects associated with ferroptosis inducers in tumor treatment, necessitating careful evaluation of their safety profiles and patient tolerability. The development of effective ferroptosis‐based therapies demands a sophisticated balance between achieving optimal therapeutic efficacy and minimizing collateral damage to healthy tissues. This equilibrium may be achieved through innovative approaches, such as advanced targeted delivery systems or strategic combinations with established therapeutic modalities. Despite encouraging preclinical results, ferroptosis inhibitors, including fer‐1, remain confined to in vitro and animal studies. The transition to human trials faces substantial obstacles, including the need for enhanced pharmacokinetic properties, improved drug stability, and mitigation of off‐target effects. Ferroptosis has emerged as a particularly promising therapeutic target in lung cancer and pulmonary diseases, offering novel intervention strategies. While research in this field continues to expand rapidly, a relatively deep understanding of the fundamental mechanisms and interrelations with the immune microenvironment is crucial. Of particular importance is the intricate crosstalk between ferroptosis and immune signaling pathways, as ferroptosis‐induced release of DAMPs and lipid peroxidation products can significantly modulate immune responses. These interactions present a double‐edged sword, potentially enhancing antitumor immunity while risking excessive inflammation and tissue damage. The dual nature of these effects underscores the need for precise modulation of ferroptosis to optimize therapeutic outcomes. Ongoing research efforts are essential to refine our understanding of these complex processes. While the comprehensive investigation of ferroptosis and its interrelation with the immune microenvironment shows promise for clinical translation, the integration of ferroptosis‐based immunotherapy into existing treatment paradigms presents significant challenges for both researchers and clinicians, particularly given the diverse array of current tumor treatment approaches. Looking forward, success in this field will require continued innovation in drug development, delivery systems, and therapeutic strategies, coupled with careful consideration of patient‐specific factors and optimal treatment combinations. This multifaceted approach will be essential in realizing the full therapeutic potential of ferroptosis‐based interventions while ensuring their safe and effective clinical implementation.

## Author Contributions

Dandan Guo and Songhua Cai prepared the figures and the manuscript, including searching the literature, writing the original draft, and editing. Wangting Xu, Jian Zhang, Lvdan Deng, Sentao Fu, Yaling Lin, Tong Jiang, Qing Li, and Zhijun Shen revised the details of this review. Peng Luo, Bufu Tang, and Ling Wang edited the manuscript. All authors have read and approved the final manuscript.

## Ethics Statement

The authors have nothing to report.

## Conflicts of Interest

The authors declare no conflicts of interest.

## Data Availability

Data sharing is not applicable to this article as no datasets were generated or analyzed during the current study.
